# Goji Disease and Pest Monitoring Model Based on Unmanned Aerial Vehicle Hyperspectral Images

**DOI:** 10.3390/s24206739

**Published:** 2024-10-20

**Authors:** Ruixin Zhao, Biyun Zhang, Chunmin Zhang, Zeyu Chen, Ning Chang, Baoyu Zhou, Ke Ke, Feng Tang

**Affiliations:** 1 School of Physics, Xi’an Jiaotong University, Xi’an 710049, China; xjtu_zrx@163.com (R.Z.); chenzeyu@opt.ac.cn (Z.C.); channingjean@163.com (N.C.); kexjtu@163.com (K.K.); fengtang@stu.xjtu.edu.cn (F.T.); 2 The Institute of Space Optics, Xi’an Jiaotong University, Xi’an 710049, China; 3 Key Laboratory for Nonequilibrium Synthesis and Modulation of Condensed Matter, Xi’an Jiaotong University, Ministry of Education, Xi’an 710049, China; 4 BA Trading (Guangzhou) Co., Ltd., Guangzhou 510000, China; lauren_zhang@live.cn; 5 Xi’an Institute of Optics and Precision Mechanics, Chinese Academy of Sciences, Xi’an 710119, China; 6 Ningxia Bing He Technology Co., Ltd., Shizuishan 753099, China; xianzby@163.com

**Keywords:** hyperspectral, diseases and pests, unmanned aerial vehicle (UAV), remote sensing monitoring

## Abstract

Combining near-earth remote sensing spectral imaging technology with unmanned aerial vehicle (UAV) remote sensing sensing technology, we measured the Ningqi No. 10 goji variety under conditions of health, infestation by psyllids, and infestation by gall mites in Shizuishan City, Ningxia Hui Autonomous Region. The results indicate that the red and near-infrared spectral bands are particularly sensitive for detecting pest and disease conditions in goji. Using UAV-measured data, a remote sensing monitoring model for goji pest and disease was developed and validated using near-earth remote sensing hyperspectral data. A fully connected neural network achieved an accuracy of over 96.82% in classifying gall mite infestations, thereby enhancing the precision of pest and disease monitoring in goji. This demonstrates the reliability of UAV remote sensing. The pest and disease remote sensing monitoring model was used to visually present predictive results on hyperspectral images of goji, achieving data visualization.

## 1. Introduction

Ningxia is a major goji berry-producing area in China, and its goji berry industry has become one of the pillars of Ningxia’s agriculture after many years of development. Goji berries have been cultivated in Ningxia for over 500 years. In recent years, as the main cash crop of the region, the planting area for goji berries has been expanding annually. The region now maintains a cultivation area of 380,000 mu (approximately 25,333 hectares), with a fresh fruit output of 300,000 tons and a total industry value exceeding 27 billion yuan. With the expansion of the cultivated area and the increase in years of cultivation, the occurrence of pests and diseases has become increasingly severe, causing significant economic losses. Common pests and diseases include the goji psyllids and the goji gall mites [1,2].

The spectral characteristics of ground objects form the basis for both theoretical and applied research in the field of remote sensing. As early as the 1980s, researchers have utilized portable spectrometers to study crop diseases and pest infestations [3]. Near-earth remote sensing spectral imaging technology involves the use of handheld or portable spectrometers to measure the spectral reflectance of crop canopies and leaves affected by diseases and pests in both laboratory and field settings. This technology is not only used for analyzing the spectral characteristics of various diseases and pests and identifying sensitive spectral bands after damage, but it also allows for the calibration of target spectral data on the ground before satellite remote sensing applications. Near-earth remote sensing spectral imaging can also determine the spectral characteristics of plants to assess the health of vegetation. Moreover, the continuous monitoring of spectral data from near-earth remote sensing spectral imaging in fields allows for the real-time tracking of crop growth, providing critical data support for scientific agricultural management.

Drone remote sensing involves the use of sensors mounted on unmanned aerial vehicles (UAVs) to gather surface information. Drones can carry high-resolution optical and multispectral sensors to capture high-resolution images of farmland, providing detailed spatial information. They can quickly cover large areas of farmland and gather real-time data, offering timely support for agricultural decision-making. The combined use of near-surface spectral technology and drone remote sensing, such as equipping drones with near-earth remote sensing instruments, can more comprehensively capture spectral information of farmland, thereby enhancing the precision of crop and soil monitoring. This is of significant importance for the management of agricultural production, pest and disease control, and the optimization of resource utilization.

In recent years, deep learning has become a pivotal tool in modern intelligent agriculture, owing to its ability to extract complex features from large datasets [4,5]. As a result, deep learning models are increasingly used in agricultural research. For example, Guo, Q et al. employed a principal component analysis and the successive projections algorithm to reduce the dimensionality of hyperspectral data for wheat rust and powdery mildew, subsequently developing discriminative models. In 2017, Whetton, R.L et al. used partial least squares regression (PLSR) to create calibration models that predict the infection rates of wheat and barley by leaf rust and fusarium diseases [6]. In 2019, Wang, X.L et al. developed a pest and disease identification model based on hyperspectral data from cotton leaves infested by aphids and spider mites using support vector machine (SVM) algorithms [7]. Similarly, in 2021, Lakshmi V et al. extracted texture features from healthy and diseased cotton leaves using the gray-level co-occurrence matrix and constructed an SVM-based pest and disease identification model [8]. In 2022, Dilixiatl, Y et al. selected specific vegetation indices to build regression models for identifying and monitoring the occurrence of cotton aphids, cotton spider mites, and cotton bollworms [9]. In the case of goji berries, pest and disease monitoring currently relies on linear methods, such as multiple regression, while research into nonlinear approaches involving neural networks remains limited [10].

## 2. Materials and Methods

### 2.1. Subsection

**Figure 1:**
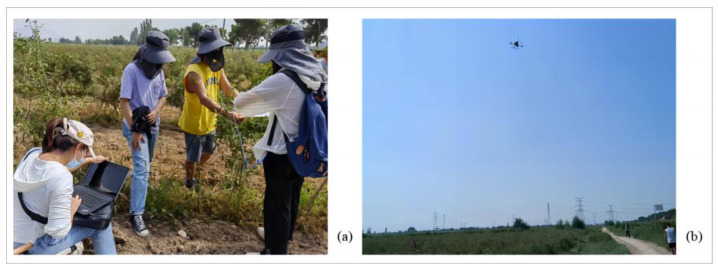
Field detection experiment diagram. (**a**) Near-ground hyperspectral detection; (**b**) UAV detection.

In the elite goji berry ecological demonstration garden located in the Dawukou District, Shizuishan City, Ningxia Hui Autonomous Region, detection was carried out using the Flame-T-VIS-NIR-ES spectrometer and the Nano-Hyperspec miniature airborne hyperspectral imager from Ocean Optics, located in Dunedin, Florida, USA. In July, when pest infestations are most common, Ningqi No. 10 goji berry cultivars in their peak fruiting phase were used as detection targets. The two experimental fields were selected under the guidance of Ms. Gao Min from the Ningxia Forest Pest Control and Quarantine Station. The fields are free of mixed pest infestations and only have single infections of either goji psyllids or goji gall mites. Each field included healthy goji plants as controls. The field detection experiment picture is shown in Figure 1. The technical specifications of the Flame-T-VIS- NIR-ES spectrometer and Nano-Hyperspec miniature airborne hyperspectral imager are detailed in Table 1 and Table 2, respectively.

### 2.2. Spectral Reflectance Measurement

Under the guidance of Ms. Gao Min from the Ningxia Forest Pest Control and Quarantine Station, the detection was performed around 13:00 on clear, sunny days with wind speeds of less than 3 on the Beaufort scale. 

Ground Spectral Data: Measurements with the handheld spectrometer were conducted with the probe positioned vertically downward and consistently about 1 m above the canopy. The field of view angle of the probe was fixed at 25°. Before each measurement, the instrument was calibrated against a standard whiteboard. To ensure the representativeness of the spectral data, branches were randomly sampled from five directions (north, south, east, west, and center) within each detection area, with 10 repeats per sample. The average value was used to represent the spectral reflectance of the goji canopy.

Drone Spectral Data: A single flight detection was conducted over the entire experimental area at a flight altitude of 15 m.

### 2.3. Data Analysis

**Figure 2:**
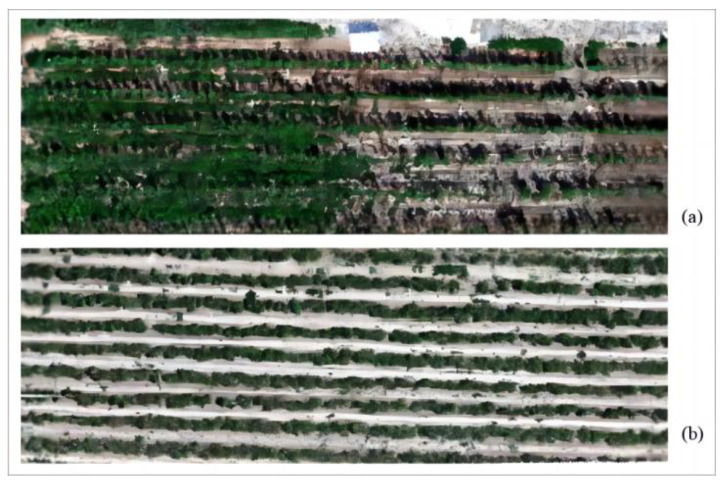
Drone images collected in Dawukou District, Shizuishan City, Ningxia Hui Autonomous Region. (**a**) Experimental field infected with goji psyllids pests; (**b**) experimental field infected with goji gall mites.

The images collected by the drone are shown in Figure 2. The experimental field infected with goji psyllids pests is shown in Figure 2a, while the field infected with goji gall mites pests is shown in Figure 2b. The collected data were processed and analyzed using ENVI 5.6, SPSS 17.0, Excel 2007, and Matlab R2022a.

#### 2.3.1. Spectral Data Preprocessing

**Figure 3:**
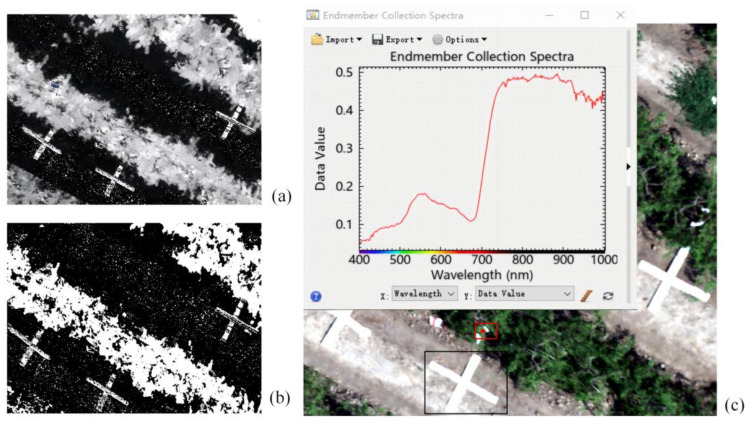
Hyperspectral image preprocessing process: (**a**) calculation of vegetation indices; (**b**) mask processing; (**c**) extraction of hyperspectral curves from the region of interest. the red box indicates the selected area of interest, the black box shows the representative goji plants marked for infection with pests and diseases.

Hyperspectral image preprocessing involves a series of steps performed on images before data analysis to enhance data quality, reduce noise, and eliminate invalid information. To mitigate the effects of background factors, outlier data are removed from the collected spectral data, and the ground spectral data are subjected to first-order derivative processing [11,12]. For the stitched drone images, noise reduction and smoothing are first performed using ENVI 5.6 software. To reduce soil interference, a masking process is applied to the drone images to segment green vegetation from the image, excluding soil and other non-target areas to enhance data quality and analytical precision. The preprocessing steps for hyperspectral images acquired by the drone are illustrated in Figure 3.

#### 2.3.2. Feature Parameter Selection

The spectral index method is an approach that utilizes spectral information from various bands in remote sensing imagery to compute specific index values, thereby reflecting the characteristics and information of the target object [13,14]. Vegetation reflectance characteristics are influenced by vegetation structure, leaf surface features, and vegetation coverage. By analyzing the reflectance across different bands, information about the growth state of the vegetation can be obtained [15,16]. The commonly used hyperspectral feature parameters in the field of remote sensing are shown in Table 3.

Vegetation indices are calculated by analyzing the light reflected from vegetation at different wavelengths to assess vegetation health, growth status, and other biological characteristics. The seven representative vegetation indices selected are shown in Table 4. 

#### 2.3.3. Method Selection

From the goji psyllid data, 6481 pixels were selected, including 98 healthy, 2064 light, 3480 middle, and 839 heavy instances. From the goji gall mite data, 2722 pixels were selected, comprising 723 healthy, 1036 light, 505 middle, and 458 heavy instances. Three methods were employed to construct remote sensing monitoring models for goji berry pests and diseases, including monadic regression (MR), multiple linear regression (MLR), and fully connected neural network (FCN), with a comparative analysis conducted thereafter. The models were validated using the coefficient of determination (R^2^) and the root mean square error (RMSE), where an R^2^ closer to 1 and a smaller RMSE indicate a better predictive performance of the model [24].

## 3. Discussion

### 3.1. Spectral Reflectance Detection Results

**Figure 4:**
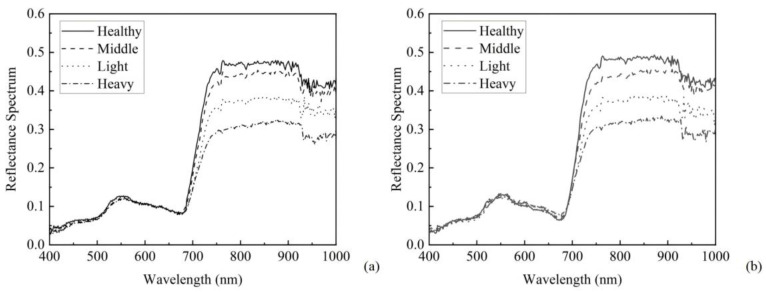
Spectral reflectance measurement results of goji canopies (**a**) for goji psyllids and (**b**) for goji gall mites.

According to the Ningxia Hui Autonomous Region Local Standard DB64/T 852-2023, plants infected with psyllids and gall mites are classified into three levels of damage: light, moderate, and severe. The canopy reflectance of goji trees infected with goji psyllids and goji gall mites are shown in Figure 4a and Figure 4b, respectively [25].

In Figure 4, it is evident that goji exhibits typical vegetation spectral characteristics, with a small reflection peak around 550 nm in the green light band. Due to chlorophyll absorption, there is strong absorption of red light, initially leading to low reflectance around 680 nm, which then increases, reaching a peak near 800 nm and reflecting strong near-infrared radiation intensely.

The canopy spectrum of psyllid-infected goji in the blue, green, and red light bands between 600 and 700 nm shows no significant difference in reflectance; however, in the red light band from 700 to 760 nm and in the near-infrared band, as the infestation level increases, the spectral reflectance decreases. This is because the nymphs and adults of psyllids adhere to the goji leaves, damaging the internal structure of the leaves, thereby reducing the reflectance in the near-infrared band.

For goji infected with gall mites, the canopy spectrum in the blue and green light bands between 500 and 550 nm shows no significant difference in reflectance. However, in the green light band from 550 to 600 nm and in the red light band from 600 to 700 nm, as the infestation level increases, the spectral reflectance rises; in the red light band from 700 to 760 nm and in the near-infrared band, as the infestation level increases, the spectral reflectance decreases. This occurs because the mites damage the leaves, turning the affected areas from green to reddish-brown and, in severe cases, to purple or black. In the visible light band, the destruction of chlorophyll in the leaves causes an increase in spectral reflectance, while in the near-infrared band, the mites cause the leaves to form patchy galls, similarly damaging the internal structure, thus reducing reflectance in this band [26].

### 3.2. Correlation Analysis of Disease Index

From Figure 4, it is evident that goji diseases and pests are sensitive to the red light band from 700 to 760 nm and the near-infrared band. Therefore, we calculate the spectral reflectance parameters for the sensitive bands at 700, 760, 850, 955, and 975 nm. 

Using SPSS 17.0 software and a Pearson two-tailed test, a correlation analysis was conducted on the hyperspectral characteristic parameters in Table 3, the vegetation indices in Table 4, and the selected sensitive bands with respect to the disease index. The results are shown in Table 5.

The results from Table 5 indicate that whether infected with psyllids or gall mites, the disease index of goji pests and diseases correlates strongly with the sum of the first-order derivative values within the blue edge, yellow edge wavelength range, or normalized value of the sum of the first-order derivatives within the red edge relative to that within the green edge, GNDVI and first-order derivative of R_700_.

### 3.3. Univariate Linear Regression Model

The construction of regression equations holds significant importance in statistical analysis. By using regression equations, one can predict the dependent variable using known values of independent variables, which is very useful for estimating and predicting future events or phenomena.

The univariate linear regression model is the simplest and most basic model in regression equation formulation [27,28]. Based on the highly significant disease index parameters in Table 5, univariate linear regression models are constructed for goji psyllids and goji gall mites. The results are shown in Table 6.

### 3.4. Multiple Linear Regression

When multiple independent variables are involved, some factors may not significantly impact the dependent variable, and there might be interactions among the independent variables, preventing them from independently influencing the dependent variable. In such cases, a stepwise regression analysis is used to filter the independent variables, identifying a subset of variables that significantly and independently affect the dependent variable [29]. Based on the highly significant disease index parameters in Table 5, multivariate stepwise regression models are constructed for goji psyllids and goji gall mites. The results are shown in Table 7.

**Figure 5:**
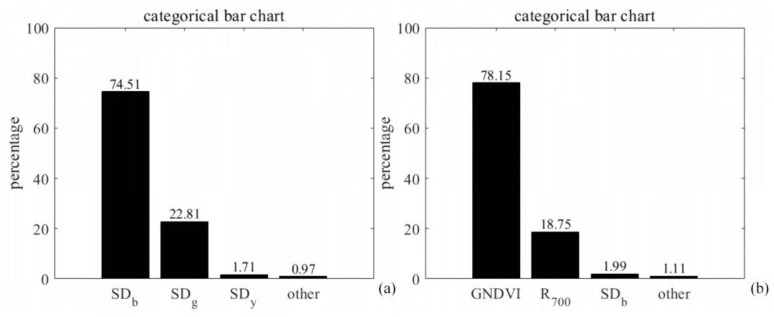
Contribution rate of a single feature parameter to the total feature parameters. (**a**) for goji psyllids and (**b**) for goji gall mites.

To verify whether multiple stepwise regression correctly eliminates the interaction between variables, a principal component analysis (PCA) was employed. PCA aims to transform data from a high-dimensional space to a lower-dimensional space through linear transformations while preserving as much variability as possible. This method allows the dataset in the lower-dimensional space to exhibit maximum variance, with the direction of maximum variance representing the linear direction of the majority of the data. The chart in Figure 5 illustrates the proportion of each eigenvalue’s contribution to the total eigenvalues (variance contribution) for the pests and diseases of goji berries related to the psyllids and gall mites.

In Figure 5, it can be observed that the cumulative contribution rates of the first principal component (F1) and the second principal component (F2) exceed 90%. This indicates that a significant amount of the original variables’ limited information has been retained, thereby allowing the construction of multiple regression models for the pest and disease of goji psyllids and goji gall mites using the first and second principal components, respectively.

### 3.5. Fully Connected Neural Network Model

Fully connected neural networks are a fundamental type of neural network architecture, in which each neuron is connected to every neuron in the previous layer. They learn patterns and relationships from input data for accurate predictions by continuously adjusting connection weights and biases and utilizing activation functions. Input signals are passed to the output layer through forward propagation, and parameters are adjusted via backward propagation to minimize prediction errors [30,31].

Fully connected neural networks (also known as dense neural networks) are a basic architecture of neural networks, in which each neuron in one layer is connected to every neuron in the next layer. Here, the fundamental mathematical principles of fully connected neural networks will be discussed through the following key components: forward propagation, loss functions, and backpropagation.

#### 3.5.1. Forward Propagation

Consider a simple fully connected neural network with one hidden layer. The network structure is as follows: input layer with *n* inputs: *x*_1_, *x*_2_, …, *x*_n_; hidden layer with *h* neurons; and output layer with *m* outputs.

1. Hidden layer: Each neuron *j* in the hidden layer receives a weighted sum of all inputs from the input layer plus a bias, as follows:
  (1) $$z_{j}=\sum_{i=1}^{n}w_{ij}x_{i}+b_{j}$$
  where *w_ij_* is the weight from input neuron *i* to hidden neuron *j*, and *b_j_* is the bias for hidden neuron *j*.
2. Apply an activation function *f* (such as ReLU) to each input of the hidden neurons to obtain the following output:
  (2) $$a_{j}=f(z_{j})$$
3. Output layer: Each neuron *k* in the output layer similarly receives a weighted sum of all outputs from the hidden layer plus a bias, as follows:
  (3) $$y_{k}=\sum_{j=1}^{h}v_{jk}a_{j}+c_{k}$$
  where *v_jk_* is the weight from hidden neuron *j* to output neuron *k*, and *c_k_* is the bias for output neuron *k*.

#### 3.5.2. Loss Functions

Commonly used loss functions include mean squared error (for regression problems) and cross-entropy loss (for classification problems).

1. The mean squared error is as follows:
  (4) $$L=\frac{1}{2}\sum_{k=1}^{m}{\left(y_{k}-t_{k}\right)}^{2}$$
  where *t_k_* is the target output.
2. The cross-entropy loss is as follows:
  (5) $$L=-\sum_{k=1}^{m}\left[t_{k}\log(y_{k})+(1-t_{k})\log(1-y_{k})\right]$$

#### 3.5.3. Backpropagation

Backpropagation is used to compute the gradient of the loss function with respect to each weight, allowing for the use of gradient descent methods to update the weights.

1. Compute the error term for the output layer as follows:
  (6) $$\delta_{k}=(y_{k}-t_{k})\cdot f^{\prime }(y_{k})$$
2. Propagate the error term to the hidden layer as follows:
  (7) $$\delta_{j}=\left(\sum_{k=1}^{m}v_{jk}\delta_{k}\right)\cdot f^{\prime }(z_{j})$$
3. Update weights and biases as follows:
  (1) Update weights in the output layer as follows:
    (8) $$v_{jk}=v_{jk}-\eta \cdot a_{j}\cdot \delta_{k}$$
  (2) Update weights in the hidden layer as follows:
    (9) $$w_{ij}=w_{ij}-\eta \cdot x_{i}\cdot \delta_{j}$$
    where *η* is the learning rate.

#### 3.5.4. Application of Fully Connected Neural Networks in the Detection of Diseases and Pests in Goji Berries

The fully connected neural network consists of several key components tailored for processing goji berry hyperspectral data. The network architecture starts with a feature input layer designed to receive 13 input features, reflecting the dimensionality of the input data. This is followed by a series of fully connected layers, each interleaved with ReLU activation layers to introduce non-linearity and aid in preventing vanishing gradients. The network culminates in a fully connected output layer with four neurons corresponding to the classification categories, topped off with a softmax layer to convert the output into a probability distribution over the predicted categories. The final piece is a classification layer, which interprets the softmax output to produce a categorical prediction.

The hidden layers of a fully connected neural network are one of the key factors influencing the performance of the network. The number of hidden layers and the number of neurons within them directly affect the model’s training and inference times. A three-layer architecture is typically a reasonable choice, as it provides sufficient flexibility and expressive capability to capture nonlinear relationships within the data. This structure can maintain high performance while avoiding excessive computational costs, thereby reducing network complexity and the risk of overfitting.

The number of neurons in the hidden layers directly determines the network’s learning capacity and performance. A higher number of neurons increases the model’s capacity to learn and express more complex patterns and features. By augmenting the number of neurons, the network can better fit the training data in a higher-dimensional space, thus enhancing model performance. However, for small datasets, an excessive number of neurons may lead to overfitting, in which the model performs well on the training set but poorly on the test set. Conversely, too few neurons may prevent the model from adequately learning the features of the training data, resulting in underfitting. Therefore, the careful selection of the number of neurons is essential to achieve good generalization ability [32,33].

**Figure 6:**
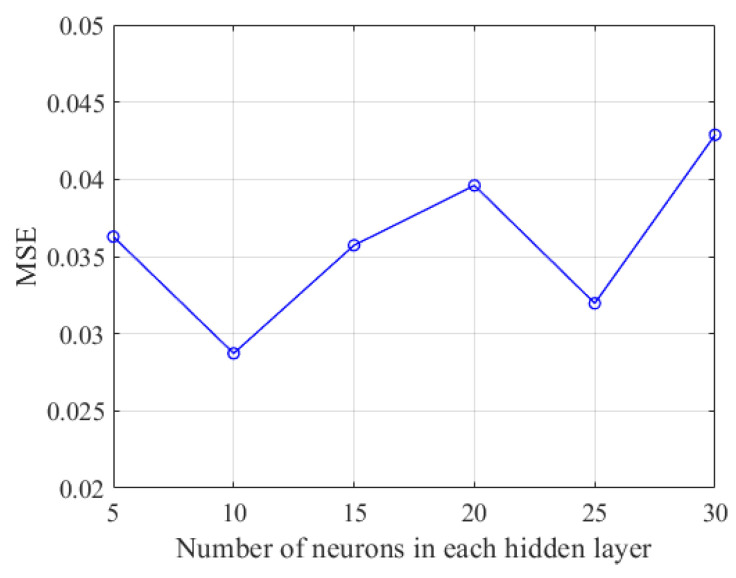
Line chart of MSE variations with changes in the number of neurons in the hidden layer.

To address this issue, the mean squared error (MSE) can be calculated to determine the optimal number of neurons in the hidden layers. A smaller MSE indicates better predictive performance. If a particular configuration yields the lowest MSE on the validation set without signs of overfitting, it is generally considered the optimal configuration. The formula for mean squared error is as follows:

(10) $$\mathrm{MSE}=\frac{1}{N}\sum_{i=1}^{N}{\left(E_{i}-P_{i}\right)}^{2}$$

where *E_i_* is the actual value, *P_i_* is the predicted value, and *N* is the total number of samples. As shown in Figure 6, when the number of neurons is set to 15, the MSE is minimized, indicating that the neural network architecture exhibits the best performance in this configuration.

## 4. Results

### 4.1. Univariate Linear Regression Model Validation

**Figure 7:**
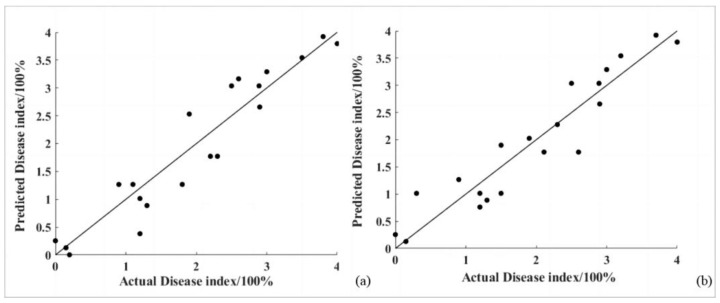
The 1:1 line plot of predicted disease indices with measured values under univariate linear regression model (**a**) for goji psyllids and (**b**) for goji gall mites.

Near-ground hyperspectral data were collected from a total of 544 samples composed of 192 samples of goji psyllids, 272 samples of goji gall mites, and 80 healthy goji samples. Twenty randomly selected near-ground hyperspectral measurements were used as a validation set, and the disease index predictions for these 20 data points were calculated using the estimated univariate linear regression models with parameter R_700′_s first derivative as the variable. A 1:1 scatter plot of the predicted versus actual values was created. Finally, the model’s performance was evaluated comprehensively using the coefficient of determination and root mean square error as metrics. The results are shown in Figure 7.

For the goji psyllid pest disease, the univariate linear regression model with parameter R_700′_s first derivative as the independent variable had a coefficient of determination of 0.8860 and a root mean square error of 0.3946.

For the goji gall mite pest disease, the univariate linear regression model with parameter R_700′_s first derivative as the independent variable had a coefficient of determination of 0.8827 and a root mean square error of 0.3860.

### 4.2. Multivariate Linear Regression Model Validation

**Figure 8:**
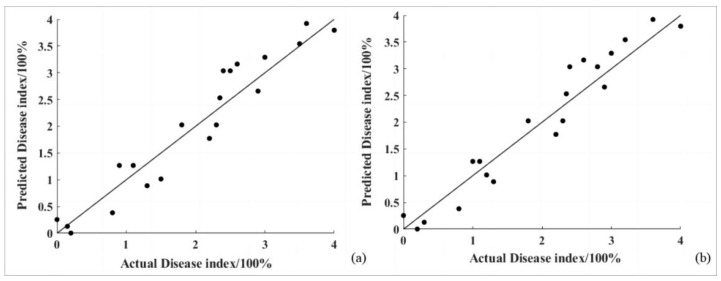
The 1:1 line plot of predicted disease indices with measured values under multiple linear regression model (**a**) for goji psyllids and (**b**) for goji gall mites.

The validation set was composed of 20 actual disease index values randomly selected from the near-ground remote sensing data. The predicted values for these 20 disease index data points were calculated using the binary linear regression model obtained in Section 4.2. A scatter plot was created to illustrate the predicted values against the actual values in a 1:1 ratio. The performance of the model was comprehensively evaluated using the coefficient of determination and the root mean square error as metrics. The results are shown in Figure 8.

The goji psyllid pest disease binary linear regression model, with SD_b_ and SD_y_ as the independent variables, had a coefficient of determination of 0.9059 and a root mean square error of 0.3531.

The goji gall mite pest disease binary linear regression model, with GNDVI and the first derivative of R_700_ as the independent variables, had a coefficient of determination of 0.9168 and a root mean square error of 0.3271.

### 4.3. Fully Connected Neural Network Model Validation

**Figure 9:**
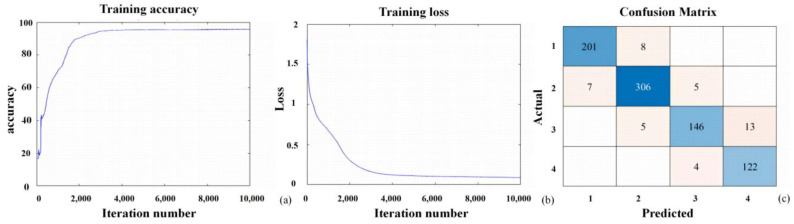
Model of goji psyllid pests and diseases constructed under a fully connected neural network: (**a**) accuracy; (**b**) loss value; (**c**) confusion matrix.The highest accuracy in the confusion matrix is represented in dark blue, while the rest are shown in light blue.

**Figure 10:**
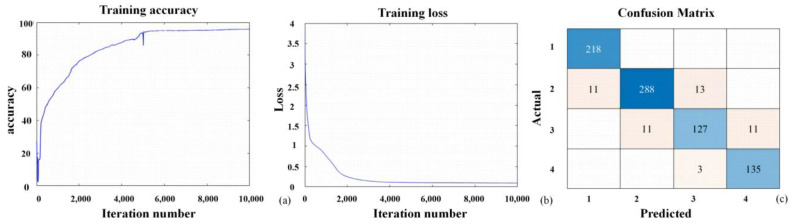
Model of goji gall mite pests and diseases constructed under a fully connected neural network: (**a**) accuracy; (**b**) loss value; (**c**) confusion matrix.The highest accuracy in the confusion matrix is represented in dark blue, while the rest are shown in light blue.

The hyperspectral image data and near-ground remote sensing data acquired by the drone were annotated based on the corresponding disease index data recorded during the experiment. The pixels associated with the tagged plants were categorized into four levels: healthy, mild, moderate, and severe, represented by the numbers 1, 2, 3, and 4, respectively. Thirteen feature parameters with the highest correlations were selected as the input layer, while the disease index (DI) served as the output layer. Three hidden layers were chosen, each containing 15 neurons. To reduce the training time of the neural network, the dataset underwent random sampling. The resulting dataset was split into training and validation sets at a ratio of 7:3, with a maximum training iteration set to 10,000 rounds to establish the neural network model. The results indicate that the validation accuracy of the fully connected neural network classification model for the goji psyllids is 94.86%, while that for the goji gall mites is 96.82%. The training accuracy, loss, and confusion matrix of the fully connected neural network model constructed for the goji psyllids and goji gall mites are shown in Figure 9 and Figure 10, respectively.

**Figure 11:**
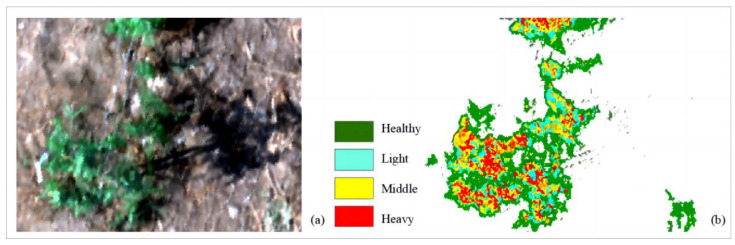
Classification results diagram of goji psyllids using a fully connected neural network. (**a**) Hyperspectral image of goji psyllids; (**b**) classification results.

Display the classification results of a randomly selected high-spectral image of goji infected with psyllid pests using fully connected neural networks. The hyperspectral image is shown in Figure 11a. Extract spectral data from each pixel of the hyperspectral image and compute its feature parameter values, preserving spatial information of the pixels. Use these feature parameters as input to the fully connected neural network, which outputs the classification results. Different colors annotate the severity of psyllid infestation on goji, visually presenting the predicted classification results onto the hyperspectral image. The classification results of the fully connected neural network are depicted in Figure 11b.

## 5. Conclusions

In the current research on monitoring diseases and pests affecting goji berries, most studies are still based on fundamental multiple linear regression models, primarily utilizing near-ground spectral remote sensing data. This paper employs a fully connected neural network to enhance the accuracy of the goji berry disease and pest prediction model, combining near-ground spectral remote sensing with drone data for the first time.

By exploring the relationships between hyperspectral feature parameters, sensitive band variables, vegetation indices, and the disease indices of the goji psyllids and goji gall mites through drone remote sensing data, highly significant correlated variables were used to construct univariate linear regression models, multiple linear regression models, and fully connected neural network models. The predictive performance of these three models was then validated using near-ground remote sensing data. The consistency between drone remote sensing and near-ground spectral remote sensing was confirmed. Due to the rapid and non-destructive detection capabilities of UAV hyperspectral technology, it holds significant promise for future agricultural crop monitoring applications.

The model based on the fully connected neural network exhibited optimal performance, achieving a prediction accuracy of 94.86% for goji psyllids and 96.82% for goji gall mites, thereby enhancing the accuracy of the goji berry disease and pest prediction model. Finally, by employing pixel-based classification, the predicted severity of goji berry diseases was directly visualized on hyperspectral images, providing a theoretical foundation and reference standard for the establishment of rapid monitoring models for goji berry diseases and pests in the field.

## Figures and Tables

**Table 1:** Parameters for handheld spectrometer.

Parameters	Value
Model	FLAME-T-VIS-NIR-ES
Signal-to-noise ratio	250:1
Spectral resolution	1.33 μm
Dark noise	50 RMS counts
Integration time	1 ms~65 s

**Table 2:** Parameters for drone-mounted hyperspectral imager.

Parameters	Value
Unmanned aerial vehicle model	DJI M300
Imaging spectrometer model	Nano-Hyperspec
Altitude	15 m
Geospatial resolution	1 cm
Lateral overlap	30%
Flight speed	1.1 m/s
Spectral wavebands	400~1000 nm
Number of channels	270
Field of view	32°

**Table 3:** Hyperspectral characteristic parameters and their descriptions.

Characteristic Parameter	Definition
SD_b_	Sum of the first-order derivative values within the blue edge wavelength range (492~530 nm)
SD_g_	Sum of the first-order derivative values within the green edge wavelength range (505~553 nm)
SD_y_	Sum of the first-order derivative values within the yellow edge wavelength range (555~571 nm)
SD_r_	Sum of the first-order derivative values within the red edge wavelength range (680~760 nm)
SD_r_/SD_b_	Ratio of the total sum of first-order derivatives within the red edge to that within the blue edge
SD_r_/SD_g_	Ratio of the total sum of first-order derivatives within the red edge to that within the green edge
(SD_r_ − SD_b_)/(SD_r_ + SD_b_)	The normalized sum of first-order derivatives within the red edge compared to that within the blue edge.
(SD_r_ − SD_g_)/(SD_r_ + SD_g_)	The normalized sum of first-order derivatives within the red edge compared to that within the green edge.
PSSR_a_	R_800_/R_680_
PSSR_b_	R_800_/R_635_
PSSR_c_	R_800_/R_470_

**Table 4:** Representative vegetation indices and their definitions.

Vegetation indices	Formula	Reference
Normalized Difference Vegetation Index	NDVI = (NIR–Red)/(NIR + Red)	[17]
Green Normalized Difference Vegetation Index	GNDVI = (NIR−Green)/(NIR + Green)	[18]
Modified Triangular Vegetation Index	MTVI = 1.2 × (1.2 × (NIR−Green) − 2.5 × (Red−Green))	[19]
Modified NDVI 705	mNDVI_705_ = (NIR−RedEdge)/(NIR + RedEdge)	[20]
Modified Simple Ratio 705	mSR_705_ = NIR/RedEdge	[21]
Red–Green Ratio Index	RGRI = Red/Green	[22]
Triangular Vegetation Index	TVI = 0.5 × (120 × (NIR−Green) − 200 × (Red−Green))	[23]

**Table 5:** Correlation analysis results between hyperspectral feature parameters and sensitive bands and the disease index of goji psyllids and goji gall mites.

Characteristic Parameter and Sensitive Bands	DI			
	Goji Psyllids		Goji Gall Mites	
	Pearson Correlation	Sig.	Pearson Correlation	Sig.
SD_b_	0.985 **	0.002	0.996 **	0
SD_g_	−0.921 *	0.026	−0.94 *	0.018
SD_y_	0.965 **	0.008	0.962 **	0.009
SD_r_	0.893 *	0.041	0.951 *	0.013
SD_r_/SD_b_	0.405	0.499	−0.149	0.811
SD_r_/SD_g_	0.753	0.142	−0.22	0.722
(SD_r_ − SD_b_)/(SD_r_ + SD_b_)	0.443	0.455	−0.051	0.935
(SD_r_ − SD_g_)/(SD_r_ + SD_g_)	0.977 **	0.004	0.975 **	0.005
PSSR_a_	0.483	0.41	0.501	0.39
PSSR_b_	0.059	0.925	0.789	0.113
PSSR_c_	0.725	0.166	0.448	0.449
GNDVI	−0.983 **	0.003	−0.99 **	0.001
first-order derivative of R_700_	−0.993 **	0.001	−0.99 **	0.001
first-order derivative of R_760_	−0.767	0.13	−0.561	0.325
R_850_	−0.945 *	0.015	0.62	0.264
R_975_/R_955_	0.89 *	0.043	0.144	0.817
NDVI	0.853	0.066	0.863	0.06
GNDVI	−0.983 **	0.003	−0.99 **	0.001
MTVI	0.508	0.382	0.56	0.326
mNDVI_705_	0.654	0.232	0.623	0.262
mSR_705_	−0.676	0.21	−0.777	0.122
RGRI	−0.533	0.355	−0.227	0.713
TVI	−0.472	0.422	−0.561	0.325

Note: “*” indicates significant correlation, while “**” indicates highly significant correlation.

**Table 6:** Univariate linear regression models of goji psyllids and goji gall mites with respect to disease index.

Types of Diseases and Pests	Simulation Equation	R^2^	Sig.
goji psyllids	DI = 10.119 − 1264.591 × first-order derivative of R_700_	0.986	0.001
	DI = 13.587 − 520.533 × SD_b_	0.967	0.003
	DI = 8.264 + 1496.754 × SD_g_	0.827	0.016
goji gall mites	DI = 5.732 − 474.858 × first-order derivative of R_700_	0.983	0.001
	DI = 5.959 − 173.345 × SD_b_	0.98	0.001
	DI = 6.072 − 155.721 × SD_g_	0.972	0.002

A higher R^2^ coefficient, closer to 1, indicates better model fit; a smaller standard error (Sig.) value indicates better regression performance. According to Table 5, the regression models using the first-order derivative of R_700_ as variables proved to be the best for both goji psyllids and goji gall mites.

**Table 7:** Multivariate stepwise regression models for the disease index of goji psyllids and goji gall mites.

Types of Diseases and Pests	Simulation Equation	R^2^	Sig.
goji psyllids	DI = 13.587 − 520.533 × SD_b_	0.976	0.003
	DI = 12.267 − 614.427 × SD_b_ − 176.924 × SD_y_	0.985	0.001
goji gall mites	DI = 5.732 − 474.858 × GNDVI	0.972	0.003
	DI = 6.223 − 544.749 × first-order derivative of R_700_ − 209.454 × GNDVI	0.988	0.001

## Data Availability

The data will be available upon request.

## References

[1] Xue Q.F., Zou G., Xie M.T. (2011). Occurrence and control measures of main pests and diseases of Goji berry. Plant Health Med..

[2] Zhang G.L. (2013). Pest and disease control techniques for Goji berry. Contemp. Hortic..

[3] Nilsson H.E. (1985). Remote Sensing of Oil Seed Rape Infected by Sclerotinia Stem Rot and Verticillium Wilt. Sver. Lantbruksuniv.

[4] Anand T., Sinha S., Mandal M. (2021). AgriSegNet: Deep Aerial Semantic Segmentation Framework for IoT-Assisted Precision Agriculture. IEEE Sens. J..

[5] Hu L.B., Lan Y.B., Zhang S.L. (2024). Research Progress on Remote Sensing Monitoring of Pests and Diseases in Cotton. Shandong Agric. Sci..

[6] Whetton R.L., Hassall K.L., Waine T.W. (2018). Hyperspectral measurements of yellow rust and fusarium head blight in cereal crops: Part 1: Laboratory study. Biosyst. Eng..

[7] Wang X.L., Deng J.Z., Huang H.S. (2019). Identification of pests incotton field based on hyperspectral data. J. South China Agric. Univ..

[8] Lakshmi V., Ramesh A., Teja Y.S. (2021). An eficient frame-work for disease detection and classification in cotton plants. J. Eng. Sci..

[9] Dilixiatl Y., Zhou J.P., Xu Y. (2022). Cotton pest monitoring based on Logistic algorithm and remote sensing image. China Agric. Univ..

[10] Wu W.L., Liu G., Ma Q. (2021). Adaptability Analysis of Aceria macrodonis Keifer in Ningxia Based on Maxent Model. Biol. J. Mt. Agric. Biol..

[11] Ge G.X. (2015). The Study of Monitoring Winter Wheat Growth and Rhizoctonia Solani Based on Remote Sensing. Master’s Thesis.

[12] Chu X.L., Yuan H.F., Lu W.Z. (2004). Progress and application of spectral data pretreatment and wavelength selection methods in NIR analytical technique. Prog. Chem..

[13] Tsai F., Philpot W. (1998). Derivative analysis of hyperspectral data. Remote Sens. Environ..

[14] Xie Y.P., Chen F.N., Zhang J.C., Zhou P., Wang H.J., Wu K.H. (2018). Study on monitoring of common diseases of crops based on hyperspectral technology. Spectrosc. Spect. Anal..

[15] Shen W.Y. (2016). Inversion of Winter Wheat Powdery Mildew Based on Hyperspectral Remote Sensing. Master’s Thesis.

[16] Pu R.L., Gong P. (2000). Hyperspectral Remote Sensing and Its Applications.

[17] Luo J., Huang W., Zhao J., Zhang J., Zhao C. (2013). Detecting aphid density of winter wheat leaf using hyperspectral measurements. Earth Obs. Remote Sens..

[18] Wang S.Q., Li W.D., Li J., Liu X.S. (2013). Prediction of soil texture using FT-NIR spectroscopy and PXRF spectrometry with data fusion. Soil. Sci..

[19] Gumma M.K. (2011). Mapping rice areas of south asia using MODIS multitemporal data. Remote Sens..

[20] Candiago S., Remondino F., De Giglio M., Dubbini M., Gattelli M. (2015). Evaluating multispectral images and vegetation indices for precision farming applications from UAV images. Remote Sens..

[21] Haboudane D. (2004). Hyperspectral vegetation indices and novel algorithms for predicting green LAI of crop canopies: Modeling and validation in the context of precision agriculture. Remote Sens. Environ..

[22] Guo R., Zhu X., Liu T. (2023). Automatic detection of crop lodging from multitemporal satellite data based on the isolation forest algorithm. Comput. Electron. Agric..

[23] Castro K., Sanchez-Azofeifa G. (2008). Changes in spectral properties, chlorophyll content and internal mesophyll structure of senescing populus balsamifera and populus tremuloides leaves. Sensors.

[24] Liang W., Li Y.J., Cen H.Y., Zhu J.P., Yin W.X., Wu W.K., Zhu H.Y., Sun D.W., Zhou W.J., He Y. (2018). Combining UAV-based vegetation indices and image classification to estimate flower number in oilseed rape. Remote Sens..

[25] Fang W., Rong Z., Wei S. (2023). Technical Regulation for Monitoring and Forecasting of Goji Insect Pests and Diseases.

[26] Ekwe M.C., Adeluyi O., Verrelst J., Kross A., Odiji C. (2024). Estimating rainfed groundnut's leaf area index using sentinel-2 based on machine learning regression algorithms and empirical models. Precis. Agric..

[27] Sims D.A., Gamon J.A. (2002). Relationships between leaf pigment content and spectral reflectance across a wide range of species, leaf structures and developmental stages. Remote Sens. Environ..

[28] Shi Y.J., Chen P.F. (2019). Maize above-ground biomass retrieval using unmanned aerial vehicle (UAV) hyperspectral remote sensing imagery. Chin. Agric. Sci. Bull..

[29] You S.B., Yan Y. (2017). Stepwise regression analysis and its application. Stat. Decis..

[30] Hornik K., Stinchcombe M., White H. (1989). Multilayer feedforward networks are universal approximators. Neural Netw..

[31] Kingma D.P., Ba J. Adam: A method for stochastic optimization. Proceedings of the 3rd International Conference on Learning Representations ICLR 2015.

[32] Tian X.J., Gong H., Tu Y. (2024). Die Casting Quality Prediction Algorithm Based on Fully Connected Neural Network. J. Netshape Form. Eng..

[33] He T., Zhou N., Wu X.Y. (2023). Thickness Prediction of Reservoir Effective Sand Body by DeepFully Connected Neural Network. J. Jilin Univ. (Earth Sci. Ed.).

